# Effects of dietary *D*-lactate levels on rumen fermentation, microflora and metabolomics of beef cattle

**DOI:** 10.3389/fmicb.2024.1348729

**Published:** 2024-02-06

**Authors:** Qian Gao, Jianfu He, Jin Wang, Yonghui Yan, Lei Liu, Zuo Wang, Weijun Shen, Fachun Wan

**Affiliations:** ^1^College of Animal Science and Technology, Hunan Agricultural University, Changsha, Hunan, China; ^2^College of Veterinary Medicine, Hunan Agricultural University, Changsha, Hunan, China

**Keywords:** beef cattle, *D*-lactate, rumen fermentation, microbiota, metabolomics

## Abstract

**Introduction:**

Excessive intake of lactate caused by improper use of silage in animal husbandry has adverse effects on rumen fermentation, such as rumen acidosis. The speed of absorption and metabolism of *D*-lactate in rumen epithelial cells was slower than that of *L*-lactate, making *D*-lactate more prone to accumulate and induce rumen acidosis. Therefore, this study was conducted to explore the effects of dietary *D*-lactate levels on rumen fermentation of beef cattle and its mechanism in an *in vitro* system.

**Methods:**

This experiment was adopted in single-factor random trial design, with 5 days for adaptation and 3 days for sample collection. Three treatments (*n* = 8/treatment) were used: (1) D-LA (0.3%), basal fermentation substrate with 0.3% (dry matter, DM basis) *D*-lactate; (2) D-LA (0.75%), basal fermentation substrate with 0.75% (DM basis) *D*-lactate; and (3) D-LA (1.2%), basal fermentation substrate with 1.2% (DM basis) *D*-lactate.

**Results:**

With the dietary *D*-lactate levels increased, the daily production of total gas, hydrogen and methane, as well as the ruminal concentrations of acetate, propionate, butyrate, isobutyrate, valerate, isovalerate, total volatile fatty acid and *D*-lactate increased (*p* < 0.05), but the ruminal pH and acetate/propionate ratios decreased (*p* < 0.05). Principle coordinate analysis based on Bray-Curtis distance showed that increasing dietary *D*-lactate levels could significantly affect the structure of rumen bacterial community (*p* < 0.05), but had no significant effect on the structure of rumen eukaryotic community (*p* > 0.05). *NK4A214_group*, *Ruminococcus_gauvreauii_group*, *Eubacterium_oxidoreducens_group*, *Escherichia-Shigella*, *Marvinbryantia* and *Entodinium* were enriched in D-LA (1.2%) group (*p* < 0.05), as well as *WCHB1-41*, *vadinBE97*, *Clostridium_sensu_stricto_1*, *Anaeroplasma* and *Ruminococcus* were enriched in D-LA (0.3%) group (*p* < 0.05). Changes in the composition of ruminal microorganisms affected rumen metabolism, mainly focus on the biosynthesis of glycosaminoglycans (*p* < 0.05).

**Discussion:**

Overall, feeding whole-plant corn silage with high *D*-lactate content could not induce rumen acidosis, and the metabolization of dietary *D*-lactate into volatile fatty acids increased the energy supply of beef cattle. However, it also increased the ruminal CH_4_ emissions and the relative abundance of opportunistic pathogen *Escherichia-Shigella* in beef cattle. The relative abundance of *Verrucomicrobiota* and *Escherichia-Shigella* may be influenced by glycosaminoglycans, reflecting the interaction between rumen microorganisms and metabolites.

## 1 Introduction

Whole-plant corn silage was the most widely used silage worldwide. The production of high-quality silage must rely on the large amount of lactate produced by lactate-producing bacteria to inhibit the proliferation of harmful bacteria and reduce feed nutrient loss, thus achieving long-term preservation (He et al., 2020). However, lactate is closely related to rumen acidosis (Nocek, 1997). Excessive intake of lactate caused by improper use of silage in animal husbandry may induce rumen fermentation dysfunction and even rumen acidosis in ruminants (Beauchemin and Yang, 2005).

At present, there is little research on the impact of exogenous lactate intake caused by the use of silage on the rumen fermentation. Previous study showed that 90% of total lactate was metabolized in the rumen, which mainly produced acetate, propionate, and butyrate (Gill et al., 1986). In addition, protozoa play an important role in ruminal lactate metabolism (Chamberlain et al., 1983). According to optical activity, lactate can be divided into *L*-lactate, *D*-lactate, and *DL*-lactate formed by mixing *L*-lactate and *D*-lactate in equal amounts. The speed of absorption and metabolism of *D*-lactate in rumen epithelial cells was slower than that of *L*-lactate, making *D*-lactate more prone to accumulate and induce rumen acidosis (Prins et al., 1974; Nagaraja and Titgemeyer, 2007). Meanwhile, because the animal body lacks the enzyme to metabolize *D*-lactate, *D*-lactate absorbed into the blood can easily accumulate and cause metabolic poisoning (Lorenz and Gentile, 2014). Since *D*-lactate is more harmful than *L*-lactate, it is necessary to study the effect of dietary *D*-lactate levels on rumen fermentation and its mechanism.

In the early stage, we measured the *D*-lactate concentration of whole-plant corn silage produced in 20 different regions of China through meteorological chromatography. The results showed that the *D*-lactate concentration ranged from 0.57 to 2.04% (DM basis; unpublished). The above result also serves as a reference for setting the *D*-lactate concentration in this study. The rumen simulation technique (RUSITEC) system is an *in vitro* fermentation device that simulates physiological functions of rumen, aiming to reduce the limitations of *in vivo* experiments, such as inconsistencies in the genetic background and physiological status of individual animals. It plays an important role in the study of ruminal microorganism and ruminal fermentation mechanism (Guo et al., 2022). Therefore, the aim of current study was to investigate the effects of dietary *D*-lactate levels on rumen fermentation and its mechanisms using RUSITEC system.

## 2 Materials and methods

### 2.1 Animals, diets, and management

Three rumen-fistulated Xiangxi yellow cattle (*Bos taurus*; a local breed in Hunan province, China) with the body weight of 385 ± 28.7 kg (mean ± SD) were used and fed a mixed diet (Table 1) of whole-plant corn silage and concentrate (6:4; DM basis) in this experiment. The basal diet of rumen fluid donor cattle was formulated according to the nutritional requirements of cattle in the Chinese Feeding Standard of Beef Cattle (NY/T815-2004). The diet was provided twice a day in 2 equal meals at 08:00 and 20:00 h.

**Table 1:** Composition and nutrient levels of the basal diets and fermentation substrate (DM basis).

Items	Content, %
Ingredients	
Whole-plant corn silage	60.00
Corn	7.40
Rice	2.00
Soybean meal	2.56
Bran	11.55
Sweet potato residues	2.40
Rice bran	3.60
DDGS	5.60
Cottonseed meal	1.92
Molasses	0.80
Limestone	1.0
Premix^a^	1.17
Total	100.00
Nutrient levels^b^	
DM	92.50
CP	12.86
EE	10.16
NDF	54.79
ADF	26.08
Ca	1.42
P	0.40

^a^ Every 1 kg of premix contained 270,000 IU of vitamin A, 30,000 IU of vitamin D_3_, 1,200 IU of vitamin E, 1.5 g of Cu, 4.5 g of Fe, 5 g of Mn, 4 g of Zn, 0.01 g of Se, 0.01 g of I, 0.01 g of Co, 55 g of Mg.

^b^ The nutrient levels were all measured values.

DDGS, distillers dried grains with solubles; DM, dry matter; EE, ether extract; NDF, neutral detergent fiber; ADF, acid detergent fiber; Ca, calcium; P, phosphorus.

### 2.2 Rumen simulation technique fermentation

The construction and operation procedures of the RUSITEC system used for this experiment were previously introduced by Arowolo et al. (2022). Briefly, the RUSITEC system is a dual-flow continuous culture system for rumen fermentation *in vitro*, which is mainly composed of fermenters, water-cooled glass containers, computer-controlled step motors, anti-blocking motors and microcontroller units. It is also equipped with a computer for automatic operation and recording, and have a signal data acquisition and program control module.

During the operation of the RUSITEC system, the temperature of the fermenter was always maintained at 39 ± 0.5°C, the McDougall’s (1948) buffer solution was continuously injected into the fermenters at a set rate through a peristaltic pump, and the overflow liquid and undegraded feed particles of each fermenter were collected into the water-cooled glass jars with a 4°C water bath. Meanwhile, the RUSITEC system could automatically and continuously discharge solids, gases, and liquids from the fermenter, which more accurately simulates rumen fermentation *in vivo*.

Before the formal test, checked the airtightness of the RUSITEC system and conducted a trial run to ensure its normal operation. Rumen contents were collected from three rumen-fistulated Xiangxi yellow cattle before morning feeding, strained through four layers of gauze into a thermos preheated at 39°C and filled with CO_2_, and taken to the laboratory. Each fermenter was filled with 500 mL strained rumen fluid and 500 mL prewarmed McDougall’s buffer solution under a constant stream of N_2_. Then 20 g of fermentation substrate (DM basis) was put into each fermenter under a stream of N_2_. The composition of fermentation substrate was consistent with the dietary composition of rumen fluid donor cattle (Table 1). The whole-plant corn silage was dried at 65°C to remove *D*-lactate from it, and then the dried whole-plant corn silage and concentrate were ground to pass a 1-mm aperture sieve and mixed at a ratio of 6:4. The temperature of the fermenter was maintained at 39 ± 0.5°C by a circulating hot water bath within a water jacket and the fermenter contents stirred by a computer-controlled stirring step motor at the rate of 25 r/min. The McDougall buffer solution was injected into each fermenter by injection pumps, maintaining the passage rate at 6%/h. The overflow liquid and small undegraded feed particle from each fermenter were collected into the water-cooled glass container maintained at a constant temperature of 4°C by circulating cold water. 20 g of fermentation substrate was put into each fermenter at 08:30 and 20:30 h daily, with N_2_ injected to maintain the anaerobic environment of the fermenter.

### 2.3 Experimental design and treatments

Twenty-four fermenters were randomly assigned to three treatments: (1) D-LA (0.3%), basal fermentation substrate with 0.3% (DM basis) *D*-lactate; (2) D-LA (0.75%), basal fermentation substrate with 0.75% (DM basis) *D*-lactate; and (3) D-LA (1.2%), basal fermentation substrate with 1.2% (DM basis) *D*-lactate. In addition, based on previous study (Ren et al., 2020), we added 1.5% (DM basis) acetate and 0.3% (DM basis) propionate into the fermentation substrate to compensate for the loss of volatile fatty acids (VFAs) in whole-plant corn silage after drying. The experimental period lasted for 8 days, consisting of 5 days for adaptation and 3 days for sample collection.

### 2.4 Sample collection

5 mL of fermentation liquid samples filtered by four layers of gauze was collected from each fermenter and placed in a 30 mL centrifuge tube before morning feeding every day. Then 10 mL of methyl green staining liquor were added into the above 30 mL centrifuge tube and shaken to rest overnight for subsequent protozoa counting. The methyl green staining liquor was obtained by dissolving 6 g of methyl green and 8 g of NaCl in 1 L of 35% formaldehyde solution. From days 5 to 8, the gas emitted from each fermenter was collected into 3 L of gas bags and 15 mL of gas samples were collected from these gas bags for the determination of methane (CH_4_) and hydrogen (H_2_) concentrations. The volume of the remaining gas in these bags was measured with a syringe with a scale line. From days 5 to 8, solid samples in water-cooled glass containers were collected in the nylon bags, cleaned, dried, and stored for determination of nutrient content. From days 5 to 8, about 10 mL of fermentation liquid samples filtered by four layers of gauze were collected from each fermenter at the 0th, 2th, 4th, 6th, 8th, 10th, and 12th h after morning feeding, with pH measured using a portable pH meter (Starter 300; Ohaus Instruments Co. Ltd.). Then the rumen liquid samples were thawed and centrifuged at 12,000 *g* for 10 min at 4°C, then 1.5 mL of supernatants were transferred into several centrifuge tubes. Among them, samples of two centrifuge tubes acidified with 0.15 mL of 25% (w/v) metaphosphoric acid and stored at −20°C for later determination of individual VFAs and NH_3_-N concentrations. The remaining samples were stored at −20°C for later determination of for subsequent determination of microbial protein (MCP), *L*-lactate and *D*-lactate concentrations. About 4 mL of fermentation liquid samples filtered by four layers of gauze were collected from each fermenter at the 4th h after the morning feeding and divided into two centrifuge tubes, immediately transferred into liquid nitrogen, and stored at −80°C for later microbiome and metabolomics analysis. And about 2 mL of fermentation liquid samples filtered by four layers of gauze were collected from each fermenter before the morning feeding, immediately transferred into liquid nitrogen, and stored at −80°C for later determination of digestive enzymes activities.

### 2.5 Chemical and biochemical analysis

The mixture of fermentation broth and methyl green liquor was dropped onto the Sedgewick-Rafter counting plate and counted under an optical microscope, following the methods described by Kisidayová et al. (2021). The analysis for CH_4_ and H_2_ concentrations of the gas samples were carried out in accordance with the previous procedures (Arowolo et al., 2022).

The DM (method 930.15), organic matter (OM, method 942.02), ether extract (EE, method 922.06) and crude protein (CP, method 988.05) of the diets and fermentation residues were analyzed according to the procedures of AOAC (2006). In addition, the calcium (Ca, method 977.29) and phosphorus (P, method 995.11) contents in the diets were detected, following the instructions of AOAC (2006). The neutral detergent fiber (NDF) and acid detergent fiber (NDF) contents in the diets and fermentation residues were determined using the methods described by previous study (Van Soest et al., 1991).

The molar concentrations of the individual VFAs were detected by gas chromatography (Chen et al., 2022) and the NH_3_-N content was assayed using the phenol-hypochlorite method (Broderick and Kang, 1980). The analysis for MCP content was carried out in accordance with the prior procedures (Makkar et al., 1982). *L*-lactate and *D*-lactate concentrations were analyzed using commercial kits (Shanghai Kexing Trading Co., Ltd., Shanghai, China) with reference to the instructions. In addition, the digestive enzymes (α-amylase, polygalacturonase, cellulase, lipase and total protease) activities of samples were detected using commercial kits (Shanghai Kexing Trading Co., Ltd., Shanghai, China) with reference to the instructions.

### 2.6 Analyses of 16S RNA and 18S RNA genes

Microbial genomic DNA was extracted from the fermentation liquid samples using E.Z.N.A. Soil DNA Kit (Omega Bio-tek, Inc., USA), following the manufacturer’s instructions. The purity and concentration of the obtained DNA was determined using TBS-380 and NanoDrop2000 spectrophotometer (Thermo Fisher Scientific, United States), respectively. And the DNA integrity was checked with 1% agarose gel electrophoresis. These DNA samples were stored at −80°C for subsequent experiments.

The rumen microbial community structure can be obtained by sequencing the region of bacterial 16S rRNA gene and eukaryotic 18S rRNA gene. The V3 and V4 hypervariable region of bacterial 16S rRNA gene were amplified with the universal primers: 338F (5′-ACTCCTACGGGAGGCAGCAG-3′) and 806R (5′-GGACTACNNGGGTATCTAAT-3′). And the V4 hypervariable region of eukaryotic 18S rRNA gene were amplified using the primers: 573F (5′-CGCGGTAATTCCAGCTCCA-3′) and 951R (5′-TTGGYRAATGCTTTCGC-3′). The purifying, quantifing and sequencing of 16S rRNA gene and 18S rRNA gene was performed on Illumina Miseq/Novaseq (Illumina, Inc., USA) platform at Beijing Allwegene Technology Co., Ltd. (Beijing, China).

Based on previous studies (Magoc and Salzberg, 2011; Chen et al., 2018), the original sequencing reads of 16S rRNA gene and 18S rRNA gene were demultiplexed, quality filtered, and merged. Qualified sequences were clustered into operational taxonomic units (OTUs) with 97% similarity using Uparse algorithm of Vsearch (v2.7.1) software, and all OTU representative sequences were classified into different taxonomic groups against Silva database using the BLAST tool. α-diversity indices (Chao1, Observed_species, PD_whole_tree and Shanon) based on the OTU information were calculated with QIIME software, and the difference test of these indices between the two groups were conducted by Wilcoxon rank-sum test. Principal coordinates analysis (PCoA) based on the Bray–Curtis distance at the OTU level was conducted for assessing β-diversity. And Adonis (PERMANOVA) analysis was performed to assess significant differences in β-diversity of bacteria and protozoa between the two groups. The taxonomic annotation and relative abundance of microbial species at phylum and genus levels were visualized as bar-plot diagrams using R (v3.6.0) software. The Wilcoxon rank-sum test within STAMP (version v.2.1.3) was used to identify differential phyla and genera between the two groups (confidence interval method). The linear discriminant analysis effect size (LEfSe) analysis was conducted by Python (v2.7) software to identify the signature microbiota between the two groups.

### 2.7 Metabolite extraction and UHPLC–MS–MS analysis

Metabolomics analysis was performed at Beijing Allwegene Technology Co., Ltd. (Beijing, China). A total of 50 μL of sample was transferred to an Eppendorf tube, and then 200 μL extraction solvent (methanol: acetonitrile = 1: 1 (v/v), containing isotopically-labeled internal standard mixture) was added. The mixture was ultrasonically treated in an ice-water bath for 10 min, followed by incubation at −40°C for 1 h to precipitate proteins. After centrifuging these samples at 4°C, 13800 *g* for 15 min, the supernatant was transferred to sample bottles for subsequent UHPLC–MS/MS analysis.

The supernatant was further separated using a UPLC system (Vanquish, Thermo Fisher Scientific), equipped with a UPLC BEH Amide column (2.1 mm × 100 mm, 1.7 μm). And the mass spectrometry data were obtained using a Q Exactive HFX mass spectrometer (Orbitrap MS, Thermal). Mobile phase was composed of part A (25 mM ammonium hydroxide and 25 mM ammonium acetate in water) and par B (100% ACN). The autosampler was set to 2 L injection volume at 4°C. The MS/MS spectra was obtained by using the QE HFX mass spectrometer with the acquisition software information-dependent acquisition (IDA) mode (Xcalibur, Thermo). The ESI conditions were as follows: sheath gas flow was 30 Arb; capillary temperature was 350°C; auxiliary gas flow was 25 Arb; MS/MS resolution was 7,500; full MS resolution was 60,000; collision energy was 10/30/60 in NCE mode; spray voltage was 3.6 kV (positive) or −3.2 kV (negative), respectively. The raw data were converted to the mzXML formats by ProteoWizard software and then processed with an in-house program, which was developed using R and based on XCMS, for identification, extraction, alignment, and integration of peak.

Principal component analysis (PCA) and orthogonal partial least squares discriminant analysis (OPLS-DA) were performed using the R package MetaboAnalystR to differentiate variables between the two groups with calculated variable importance in projection (VIP) value. Unsupervised PCA exhibited the distribution of origin data, and supervised OPLS-DA were applied to attain a higher level of group separation and acquire a better understanding of variables responsible for classification. Then parameters R2Y and Q2 were calculated, which represented the explanatory power and predictive power of the OPLS-DA model, respectively. To avoid overfitting of the OPLS-DA model, 200 times permutation was further conducted. The metabolites with VIP values >1 and *p* < 0.05 (Student’s t-test) and fold change (FC) > 1.5 or < 0.67 were screened as significantly differential metabolites. These metabolites were annotated using KEGG,^1^ and the annotated metabolites were selected through MetaboAnalyst^2^ to search for potential metabolic pathways.

### 2.8 Statistical analysis

Statistical analyses were subjected to one-way ANOVA using SPSS 20.0 software (SPSS, Inc., Chicago, IL, USA), and expressed as means ± SEM. Duncan’s multiple range test was used to detect the significance of differences among treatment means. The Graphpad Prism 8.0 software (Origin, CA, USA) was used for visualization of partial results. Spearmen’s rank correlation tested the relationships between the differential metabolites and microorganisms (genus level). Statistical difference was, respectively, regarded as highly significant or significant at *p* < 0.01 or *p* < 0.05.

## 3 Results

### 3.1 Effects of dietary *D*-lactate levels on the rumen protozoa count of beef cattle

**Figure 1:**
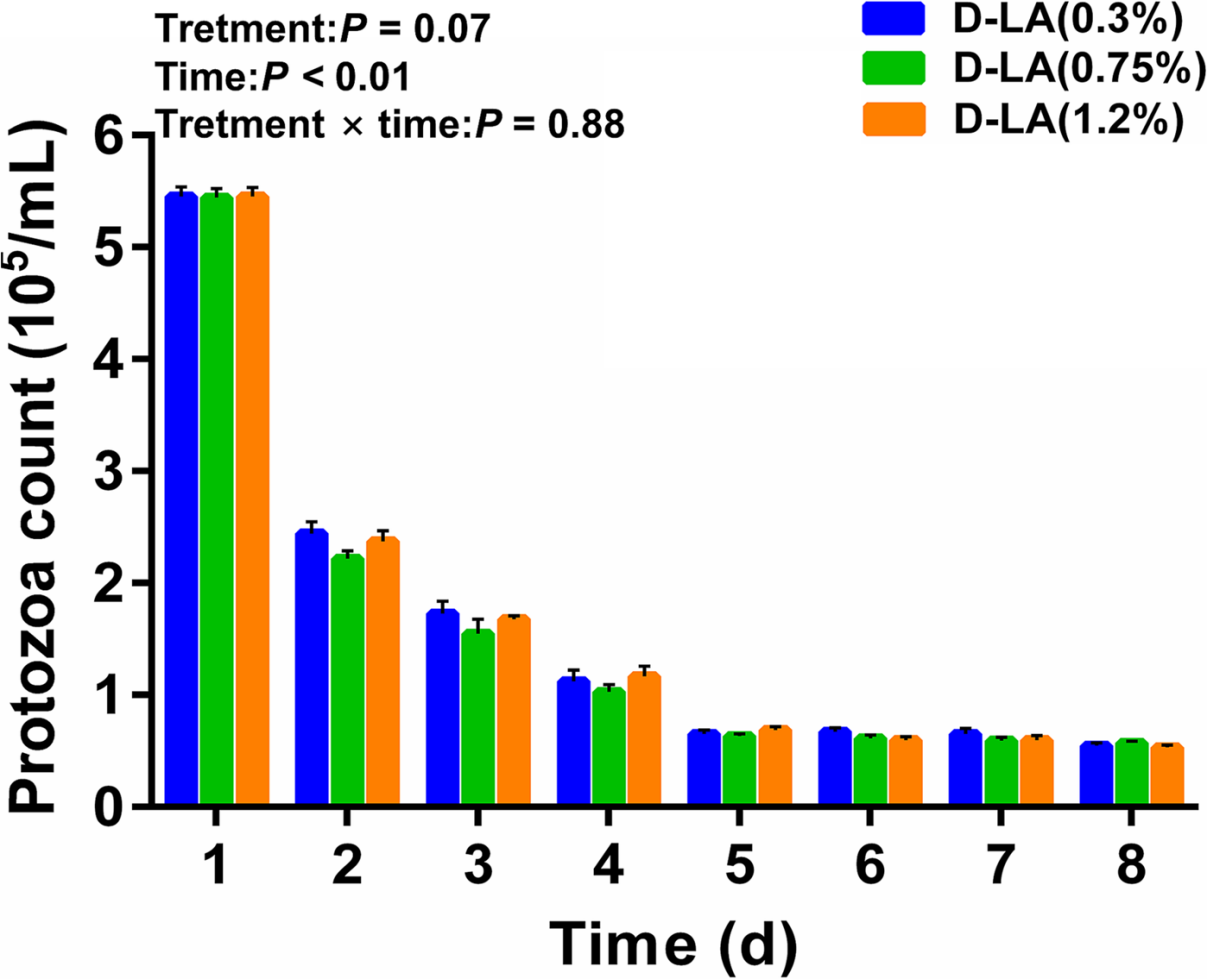
Dynamic changes of the rumen protozoa count in the three groups from days 1–8.

As shown in Figure 1, the rumen protozoa count of the three groups decreased rapidly between days 0 and 1, decreased slowly between days 2 and 4, and tended to be stable between days 5 and 8. The rumen protozoa count was not affected by dietary *D*-lactate levels (*p* > 0.05), but decreased with the increase of sample days (*p* < 0.05). There was no significant interaction between the dietary *D*-lactate levels and sampling days on the rumen protozoa count (*p* > 0.05).

### 3.2 Effects of dietary *D*-lactate levels on nutrient disappearance rates, digestive enzymes activities and gas production in the rumen of beef cattle

As shown in Table 2, the nutrient (DM, NDF, ADF, OM, CP and EE) disappearance rates and the digestive enzymes (α-amylase, polygalacturonase, cellulase, lipase and total protease) activities were not affected by dietary *D*-lactate levels (*p* > 0.05). However, with the increase of dietary *D*-lactate levels, the daily total gas, H_2_ and CH_4_ production increased (*p* < 0.05).

**Table 2:** Effects of dietary *D*-lactate levels on nutrient disappearance rates, digestive enzymes activities and gas production in the rumen of beef cattle.

Items	Dietary *D*-lactate levels, DM basis			SEM	*p*-value		
	0.3%	0.75%	1.2%		Treatment	Linear	Quadratic
Nutrient disappearance rates (%)							
DM	80.39	80.10	79.95	0.33	0.87	0.60	0.93
NDF	69.24	68.35	69.35	0.42	0.59	0.92	0.31
ADF	61.85	60.68	60.50	0.44	0.42	0.23	0.60
OM	79.11	78.74	78.80	0.25	0.82	0.63	0.70
CP	81.54	80.91	81.30	0.20	0.47	0.64	0.26
EE	84.46	84.04	84.10	0.18	0.61	0.44	0.54
Digestive enzymes activities							
α-amylase (U/L)	224.01	243.09	230.89	4.58	0.24	0.54	0.11
Polygalacturonase (U/L)	2.11	2.19	1.91	0.07	0.26	0.25	0.24
Cellulase (U/mL)	429.03	412.33	463.53	12.77	0.26	0.27	0.22
Lipase (U/L)	122.14	115.98	114.45	2.56	0.45	0.24	0.68
Total protease (U/L)	38.55	39.91	41.11	0.79	0.44	0.20	0.96
Gas production							
Total gas (L/d)	6.40^c^	6.60^b^	6.88^a^	0.54	<0.01	<0.01	0.63
H_2_ (mL/d)	9.98^b^	10.75^b^	11.71^a^	0.23	<0.01	<0.01	0.80
CH_4_ (mL/d)	887.31^b^	927.18^a^	950.15^a^	8.00	<0.01	<0.01	0.53

^a–c^ Values with in a row without common letters differ significantly (*p* < 0.05).

SEM, standard error of the mean; DM, dry matter; NDF, neutral detergent fiber; ADF, acid detergent fiber; OM, organic matter; CP, crude protein; EE, ether extract.

### 3.3 Effects of dietary *D*-lactate levels on dynamic changes in the ruminal fermentation parameters during 12 h after morning feeding

**Figure 2:**
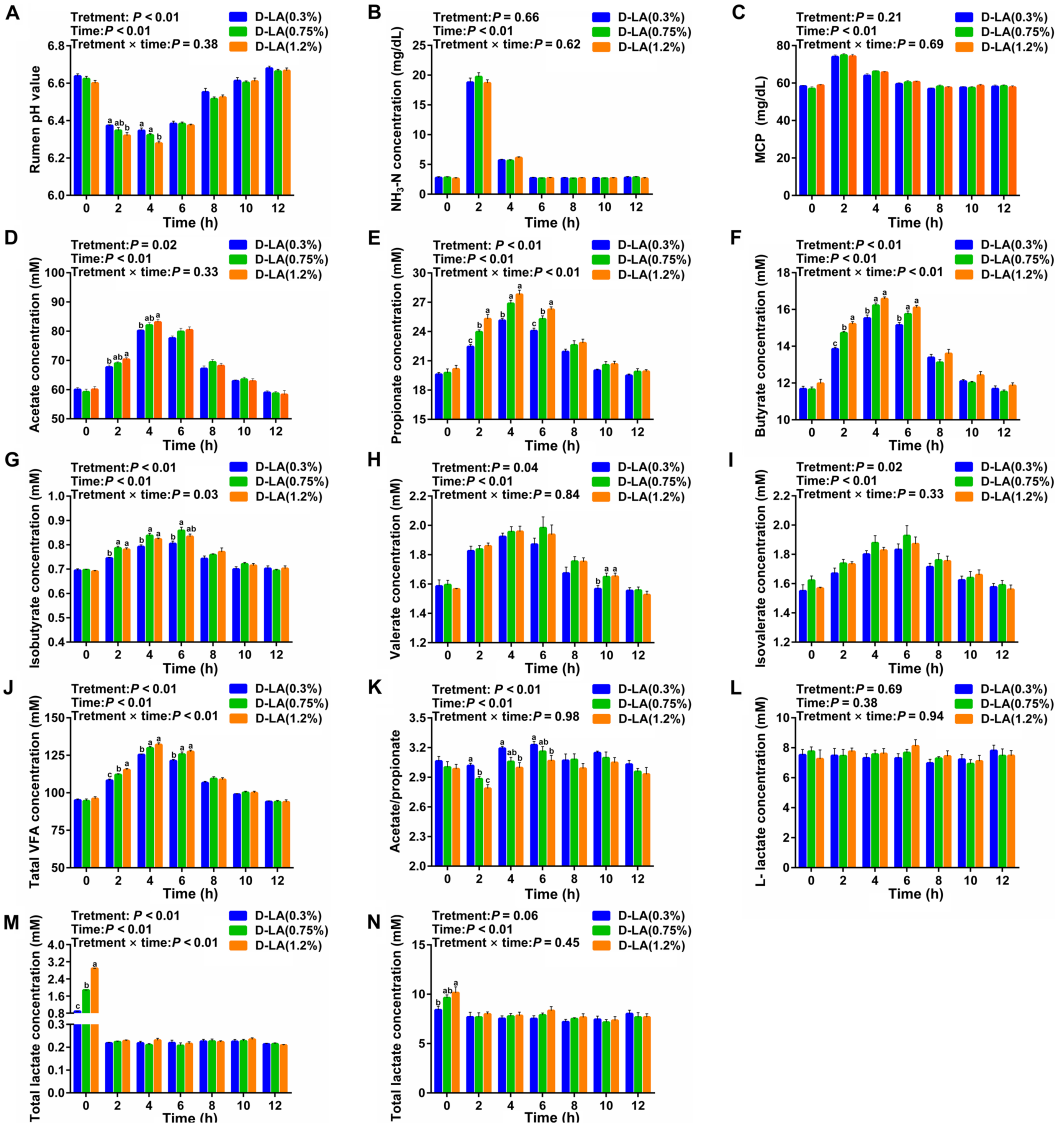
Dynamic changes in ruminal fermentation parameters of beef cattle within 12 h after feeding. Dynamic changes in the ruminal **(A)** pH value, **(B)** NH_3_-N concentration, **(C)** MCP concentration, **(D)** acetate concentration, **(E)** propionate concentration, **(F)** butyrate concentration, **(G)** isobutyrate concentration, **(H)** valerate concentration, **(I)** isovalerate concentration, **(J)** TVFA concentration, **(K)** acetate/ propionate ratios, **(L)** *L*-lactate concentration, **(M)** *D*-lactate concentration, and **(N)** total lactate concentration. Data were shown as means ± SEM (*n* = 8). Values of each parameter with in a histogram at a certain time point with different small letter superscripts mean significant difference (*p* < 0.05). For each index, bars without sharing a common letter indicated significant differences (*p* < 0.05).

Dynamic variations of ruminal fermentation parameters from one feeding time point to another adjacent feeding time point for a total of 12 h were displayed in Figure 2. Overall, increasing dietary *D*-lactate levels increased ruminal acetate, propionate, butyrate, isobutyrate, valerate, isovalerate, TVFA and *D*-lactate concentrations (*p* < 0.05), but decreased the ruminal pH and acetate/propionate ratios (*p* < 0.05). The results also showed that all fermentation parameters except for *L*-lactate concentration were affected by sampling time (*p* < 0.05). There was a significant interaction between the treatments and sampling time on the ruminal propionate, butyrate, isobutyrate, TVFA and *D*-lactate concentrations (*p* < 0.05).

Specifically, ruminal pH in the D-LA (1.2%) group was lower (*p* < 0.05) than that in the D-LA (0.3%) group at the 2th h, and ruminal pH in the D-LA (1.2%) group was lower (*p* < 0.05) than that in other two groups at the 4th h (Figure 2A). At the 2th and 4th h, ruminal acetate concentration in the D-LA (1.2%) group was significantly higher than that in the D-LA (0.3%) group (Figure 2D). Ruminal propionate concentration increased (*p* < 0.05) with the dietary *D*-lactate levels increased at the 2th and the 6th h, and the propionate content in the D-LA (0.3%) group were lower (*p* < 0.05) than that in other two groups at the 4th h (Figure 2E). An increase (*p* < 0.05) in butyrate and TVFA concentrations was observed in response to the increasing dietary *D*-lactate levels at the 2th h, and ruminal butyrate and TVFA concentrations in the D-LA (0.3%) group were lower (*p* < 0.05) than that in other two groups at the 4th and 6th h (Figures 2F,J). Isobutyrate content in the D-LA (0.3%) group was lower (*p* < 0.05) than that in other two groups at the 2th and 4th h, and isobutyrate content in the D-LA (0.3%) group was lower (*p* < 0.05) than that in the D-LA (0.75%) group at the 6th h (Figure 2G). Valerate content in the D-LA (0.3%) group was lower (*p* < 0.05) than that in other groups at the 10th h (Figure 2H). Acetate/propionate ratios decreased (*p* < 0.05) with the increase of dietary *D*-lactate levels at the 2th h, and acetate/propionate ratios in the D-LA (0.3%) group were higher (*p* < 0.05) than that in the D-LA (1.2%) group at the 4th and 6th h (Figure 2K). An increase (*p* < 0.05) in *D*-lactate and total lactate concentrations was observed in response to the increasing dietary *D*-lactate levels at the 0th h (Figures 2M,N).

### 3.4 Effects of dietary *D*-lactate levels on rumen microbiota

#### 3.4.1 Effects of dietary *D*-lactate levels on the composition and diversity of rumen bacterial community

**Figure 3:**
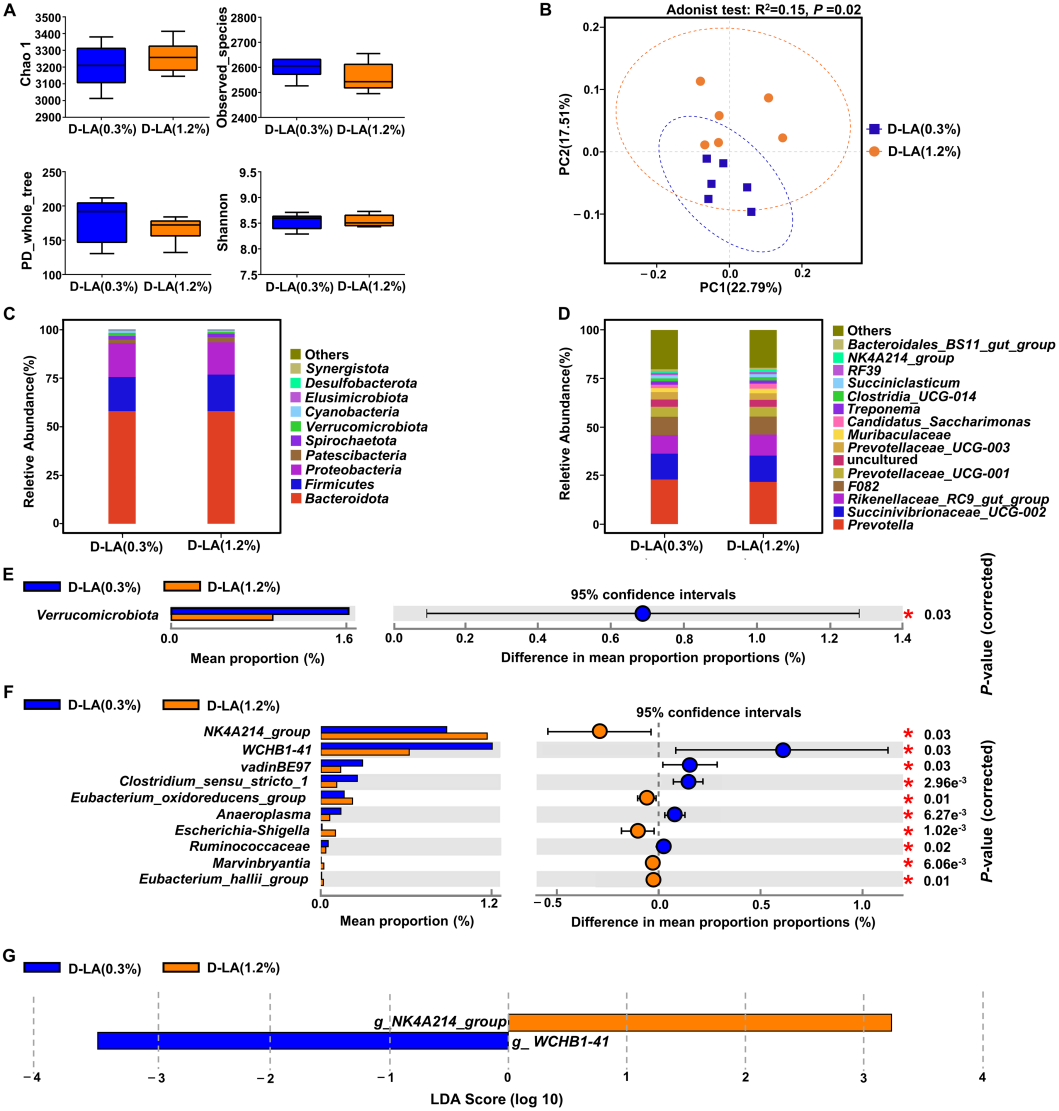
Effects of dietary *D*-lactate levels on the composition and diversity of rumen bacterial community (*n* = 6). **(A)** α-diversity indices, including Chao 1, Observed_species, PD_whole_tree and Shannon. **(B)** β-diversity indicated by PCoA based on Bray-Curtis distance. The composition of rumen bacterial community of beef cattle at the **(C)** phylum and **(D)** genus levels. STAMP difference analysis of rumen bacterial community between the two groups at the **(E)** phylum and **(F)** genus level (top 10). **(G)** Bar charts showing LDA scores across treatments. Significant differences were defined as *p* < 0.05 and LDA score > 3.0.

As shown in Figure 3A, there was no significant difference between the two groups in the α-diversity indices (Chao1, Observed_species, PD_whole_tree and Shannon) of the bacterial community (*p* > 0.05). However, PCoA based on Bray-Curtis distance and Adonis test (Figure 3B) revealed that dietary *D*-lactate levels had a significant effect on the composition of ruminal bacteria (*p* < 0.05). At the phylum level, *Bacteroidota*, *Firmicutes* and *Proteobacteria* were the dominant bacteria in the two groups (Figure 3C). The relative abundance of *Verrucomicrobiota* was lower (*p* < 0.05) in the D-LA (1.2%) group than that in the D-LA (0.3%) group (Figure 3E). At the genus level, the relative abundance of *Prevotella*, *Succinivibrionaceae_UCG-002*, *Rikenellaceae_RC9_gut_group* were the dominant bacteria in the two groups (Figure 3D). The relative abundance of *WCHB1-41*, *vadinBE97*, *Clostridium_sensu_stricto_1*, *Anaeroplasma* and *Ruminococcus* in the D-LA (1.2%) group decreased (*p* < 0.05) as compared with those in the D-LA (0.3%) group (Figure 3F). In contrast, compared with the D-LA (0.3%) group, the relative abundance of *NK4A214_group*, *Eubacterium_oxidoreducens_group*, *Escherichia-Shigella*, *Marvinbryantia* and *Eubacterium_hallii_group* in the D-LA (1.2%) group decreased (*p* < 0.05) (Figure 3F).

The linear discriminant analysis effect size (LEfSe) analysis (Figure 3G) was performed to identify the differential microbiota that varied with dietary *D*-lactate levels. With a default LDA cutoff ±3, differential taxa totaling 1, and 1 in the D-LA (0.3%) group and D-LA (1.2%) group, respectively. The bacterial biomarkers in the D-LA (0.3%) group was *WCHB1-41*, and in the D-LA (1.2%) group was *NK4A214_group*.

#### 3.4.2 Effects of dietary *D*-lactate levels on the composition and diversity of rumen eukaryotic community

**Figure 4:**
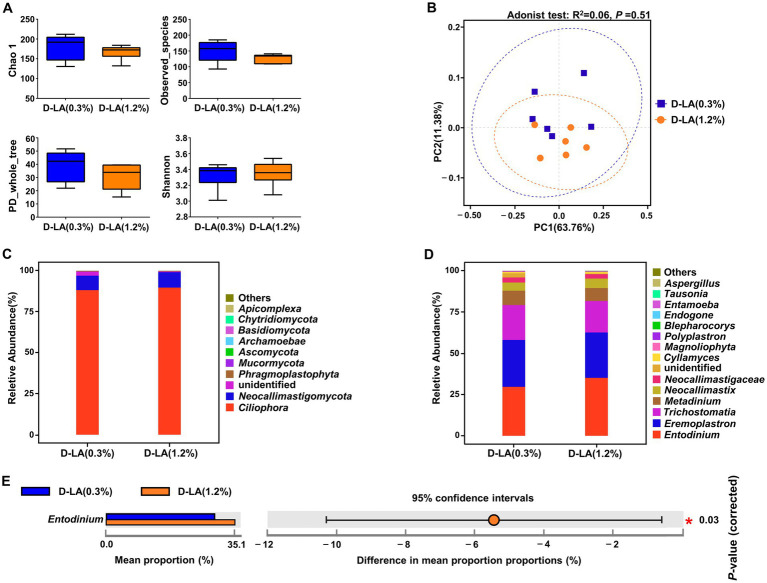
Effects of dietary *D*-lactate levels on the composition and diversity of rumen eukaryotic community (*n* = 6). **(A)** α-diversity indices, including Chao1, Observed_species, PD_whole_tree and Shannon. **(B)** β-diversity indicated PCoA based on Bray-Curtis distance. The composition of rumen eukaryotic community at the **(C)** phylum and **(D)** genus levels. **(E)** STAMP difference analysis of rumen eukaryotic community between the two groups at the genus level.

As shown in Figures 4A,B, there was no significant difference in the α-diversity and β-diversity of the eukaryotic community between the two groups (*p* > 0.05). At the phylum level, *Ciliophora* was the dominant eukaryotes in the two groups (Figure 4C). There was no differential phylum between the two groups (*p* > 0.05). At the genus level, *Entodinium*, *Eremoplastron*, and *Trichostomatia* were the dominant eukaryotes in the two groups (Figure 4D). And the relative abundance of *Entodinium* in the D-LA (1.2%) group decreased (*p* < 0.05) as compared with that in the D-LA (0.3%) group (Figure 4E).

### 3.5 Effects of dietary *D*-lactate levels on the rumen metabolites

**Figure 5:**
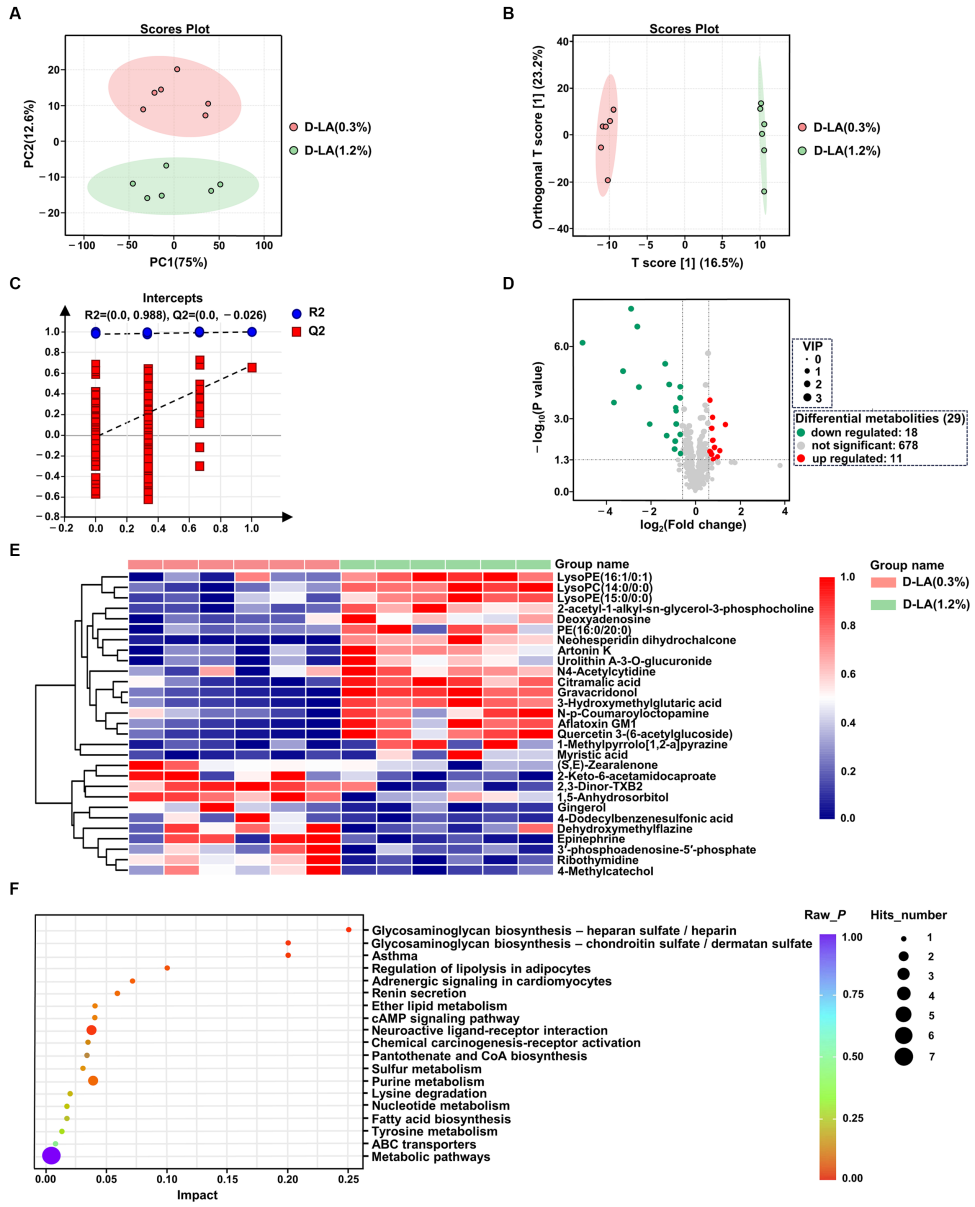
Metabolites analysis of rumen fluid in the two groups by untargeted metabolic profiling (*n* = 6). **(A)** PCA of the ruminal metabolites between the two groups. **(B)** OPLS-DA of the ruminal metabolites between the two groups. **(C)** Permutation test of OPLS-DA. **(D)** Volcano map of metabolites. **(E)** Cluster analysis of differential metabolites between the two groups. **(F)** KEGG pathway enrichment analysis of differential metabolites between the two groups.

The UPLC-MS/MS platform was used to analyze the changes of rumen metabolites. After quality control, a total of 707 ruminal metabolites were identified and quantified in positive ion mode and negative ion mode. Principal component analysis (PCA) score plot was performed to visualize the overall differences of metabolites between the two groups. In PCA score plots (Figure 5A), the distance separating symbols between the two groups was much greater than that within groups, which indicated that significant differences in metabolites between the two groups (*p* < 0.05). The metabolite disturbances were characterized using the orthogonal partial least squares discriminant analysis (OPLS-DA) to further identify differences in metabolites between the two groups. The OPLS-DA score plots (Figure 5B) showed that there were differences in the distribution between the two groups. Both R2Y and Q2 values of the OPLS-DA model were greater than 0.5 (Supplementary Figure S1), indicating that this model had a good degree of reliability and predictive ability. In addition, 200 random permutations and combination testing was used to verify the accuracy of the model. As shown in Figure 5C, both the R2 and Q2 values were lower than initial values, demonstrating that this model was not overfitted (Fu et al., 2023).

OPLS-DA model derived VIP scores ≥1.0, *p* < 0.05 in Student’s t-test, and FC values >2 or < 0.67 were used as criterion to screen out differentially expressed metabolites. Volcano plots were used to visually reveal 29 differential metabolites between the two groups, including 18 down-regulated and 11 up-regulated metabolites (Figure 5D and Supplementary Table S1). A further understanding of how ruminal metabolites change with increasing dietary *D*-lactate levels is essential. Therefore, hierarchical clustering analysis was performed for the above 29 differential metabolites. As shown in Figure 5E, samples of the two groups were mainly concentrated in two clusters. Compared with the D-LA (0.3%) group, metabolites that significantly decreased or increased in the D-LA (1.2%) group were separated clearly. Furthermore, the KEGG pathway enrichment analysis was performed to investigate the relevant metabolic pathways affected by dietary *D*-lactate levels. The above 29 differential metabolites were mainly enriched in 19 metabolic pathways. Five of the nineteen metabolic pathways metabolic pathways were differential between the two groups (*p* < 0.05), including glycosaminoglycan biosynthesis – chondroitin sulfate / dermatan sulfate, glycosaminoglycan biosynthesis – heparan sulfate / heparin, asthma, adrenergic signaling in cardiomyocytes, and neuroactive ligand-receptor interaction (Figure 5F).

### 3.6 Correlation analysis between the rumen differential microorganisms and metabolites

**Figure 6:**
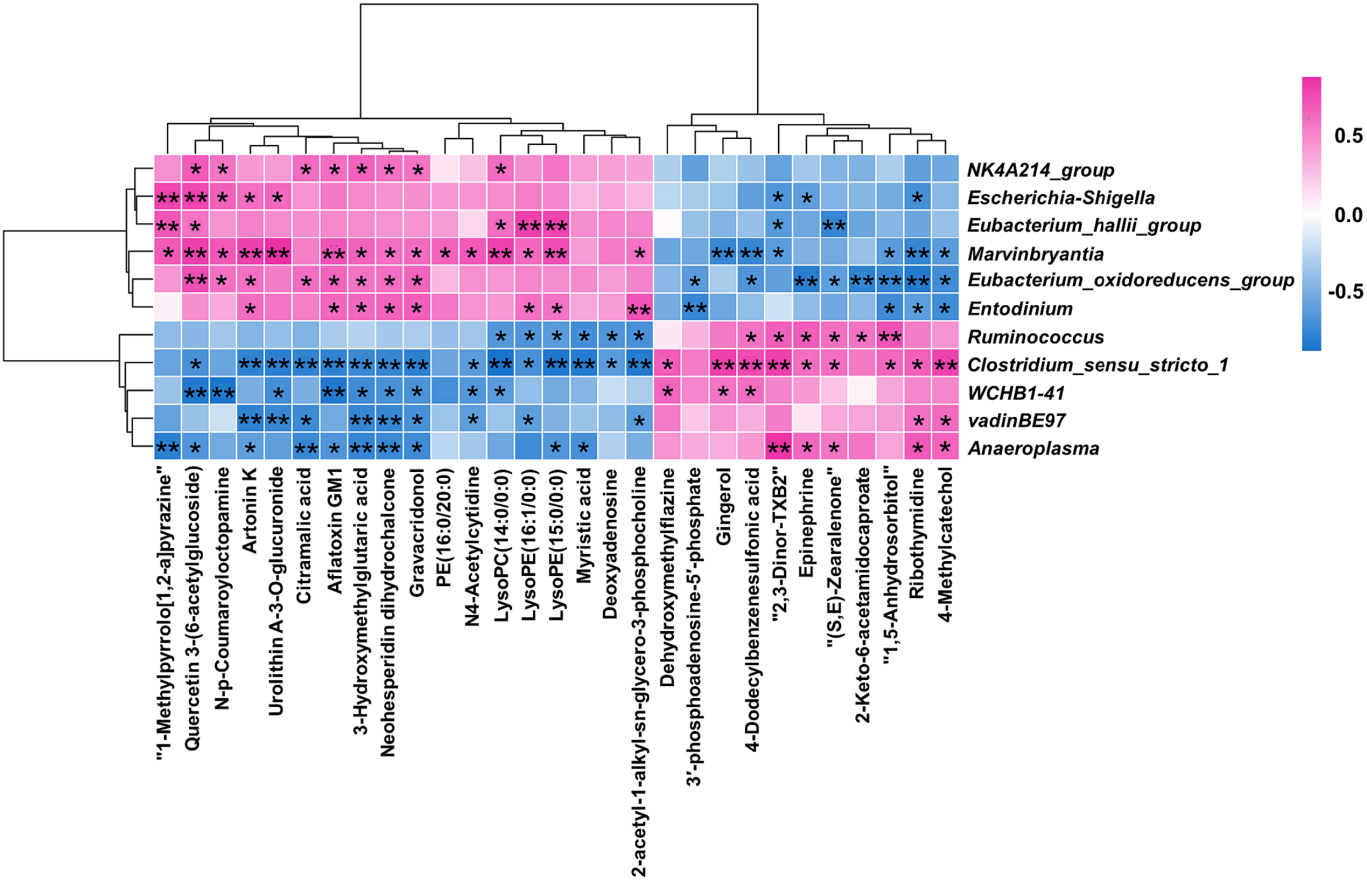
Statistical Spearman’s correlations between the differential microorganisms and metabolites. Red represented positive correlation, and blue represented negative correlation. ** indicated *p* < 0.01, and * indicated *p* < 0.05.

A Spearman rank correlation tested the relationships between the 11 differential microorganisms (genus level) and the 29 differential metabolites (Figure 6). There were complex interactions between the ruminal microbiota and metabolites, with each differential microorganism significantly correlated with at least seven differential metabolites (|r| > 0.6; *p* < 0.05). 2-acetyl-1-alkyl-sn-glycero-3-phosphocholine, Epinephrine, and 3′-phosphoadenosine-5′-phosphate were enriched in four differential metabolic pathways between the two groups. *Entodinium*, *Clostridium_sensu_stricto_1*, *Eubacterium_oxidoreducens_group* and *Ruminococcus* were significantly (|r| > 0.6 and *p* < 0.05) correlated with two of the three differential metabolites mentioned above. The above results indicated that *Entodinium*, *Clostridium_ Sensu_ Stricto_ 1*, *Eubacterium_ oxidoreducens_ group* and *Ruminococcus* may play a crucial role in *D*-lactate mediated changes in ruminal metabolism.

## 4 Discussion

The stability of the RUSITEC system during operation is related to the accuracy and reliability of experimental data. Protozoa, as the largest rumen microbiota, are easy to be observed under a microscope. Therefore, we preliminarily evaluated the operational status of the RUSITEC system by counting ruminal protozoa every day. In this experiment, the protozoa count rapidly decreased between days 1 and 5, and tended to be stable between days 6 and 8 (Figure 1 and Supplementary Table S2), which was consistent with previous study (Li et al., 2023). Therefore, it is reasonable for us to conduct sampling between days 6 and 8. Total gas production is an important indicator for evaluating rumen fermentation, which is positively correlated with the degradation degree of fermentation substrate (Krieg et al., 2017). In the current study, the total gas production increased with the increase of dietary *D*-lactate levels, but the nutrient disappearance rates were not affected by dietary *D*-lactate levels. This phenomenon may be due to the metabolism of *D*-lactate to VFAs accompanied by gas generation, rather than an increase in the degradation degree of fermentation substrate. H_2_ is an important intermediate in the production of acetyl-CoA from the reduction equivalent released by oxidative decarboxylation of pyruvate during rumen fermentation (Lourenço et al., 2010). Therefore, the increased dietary *D*-lactate may be metabolized to produce more pyruvate, leading to an increase in H_2_ through increasing the oxidative decarboxylation of pyruvate. Moreover, H_2_ can be utilized by methanogens to produce CH_4_ (Du et al., 2018). This could partly explain the increase in CH_4_ production with increasing of dietary *D*-lactate levels.

Rumen pH plays an important role in maintaining the growth and metabolism of rumen microorganisms. The rumen pH value in the range of 6.0 to 7.0 indicates that the rumen is in a healthy status (Calsamiglia et al., 2002). In this study, the ruminal pH values of the three groups ranged from 6.2 to 6.8, indicating that increasing dietary *D*-lactate levels had no negative affect on the ruminal health status. Moreover, increasing dietary *D*-lactate levels reduced ruminal pH, which mainly due to the increase of acetate, propionate, butyrate, isobutyrate and total VFA concentrations. It is worth noting that lactate-to-VFAs conversion could promote the ruminal buffering, which is an important mechanism to prevent ruminal acidosis (Penner et al., 2009). Acetate could be utilized to produce CH_4_ by acetotrophic methanogens (Liu et al., 2001). It could be thus inferred that the increase in acetate concentration and H_2_ production with the increase of dietary *D*-lactate levels may jointly lead to an increase in CH_4_ production in this study. In this trial, the decrease of acetate/propionate ratios indicated that increasing dietary *D*-lactate levels could improve the energy supply of beef cattle by changing rumen fermentation pattern. In addition, *D*-lactate could be rapidly metabolized to a relatively stable level after morning feeding, which was consistent with previous study (Chamberlain et al., 1983).

In the present work, we illustrated the effects of increasing dietary *D*-lactate levels on the taxonomic composition of rumen bacteria and eukaryotes. At the phylum level, *Verrucomicrobiota* was the only differential bacteria between the two groups, and there was no differential eukaryote between the two groups. *Verrucomicrobiota* contains highly specialized species degrading complex polysaccharides, such as *WCHB1-4* (van Vliet et al., 2019). In this study, *Verrucomicrobiota* was enriched in the D-LA (0.3%) group, indicating that increasing dietary *D*-lactate levels may have adverse effects on polysaccharide degradation in the rumen. At the genus level, *Escherichia-Shigella* is an opportunistic bacterium that can cause infection of the gastrointestinal tract (Suh et al., 2014). The relative abundance of *Escherichia-Shigella* in the D-LA (1.2%) group was higher than that in the D-LA (0.3%) group, suggesting that increasing dietary *D*-lactate levels could increase the risk of infection of the gastrointestinal tract in beef cattle. In addition, *Escherichia-Shigella* can metabolize formate into CO_2_ and H_2_ to obtain energy (Yoshida et al., 2005). Lactate is catalyzed by lactate dehydrogenase to produce pyruvate, and a portion of pyruvate can be catalyzed by pyruvate formate-lyase to form formate in rumen. It could be thereby assumed that increasing dietary *D*-lactate levels may increase ruminal formate content to provide more energy for *Escherichia-Shigella*, ultimately leading to the increase in the relative abundance of *Escherichia-Shigella*, H_2_ production and total gas production. With the exception of *vadinBE97*, *WCHB1-41* and *Escherichia-Shigella*, the other seven differential bacteria belong to *Firmicutes*. In addition, *Entodinium* was the most dominant protozoan genus in the two groups, and the only one differential protozoan genus between the two groups. It plays an important role in the generation of ruminal CH_4_ (Ranilla et al., 2007). Therefore, *Entodinium* may make an important contribution to the increase in H_2_ and CH_4_ production caused by increasing dietary *D*-lactate levels. Previous study showed that the relative abundance of methanogens was not always positively correlated with ruminal CH_4_ production (Danielsson et al., 2012). This may explain that there was no significant difference in the relative abundance of methanogens between the two groups in this study. The main metabolites are different in different species, so changes in the composition of microbial community may affect the molar proportion of individual VFAs in rumen (Saleem et al., 2013). *NK4A214*, *Eubacterium_ Oxidoreducens_ group*, *Eubacterium_ hallii_ group* and *Marvinbryantia* mainly produce butyrate (Hold et al., 2002; van der Beek et al., 2017; Mukherjee et al., 2020; Verhoeven et al., 2021), *Clostridium_ Sensu_ Stricto_ 1*, *Anaeroplasma* and *Ruminococcus* mainly produce acetate (Lee et al., 2020; Lu et al., 2020; Chen et al., 2021), as well as *Entodinium* could phagocytose starch and greatly contribute to the production of propionate (Ivan et al., 2000). Therefore, increasing dietary *D*-lactate levels increased the relative abundance of butyrate-producing and propionate-producing microorganisms, but decreased the relative abundance of acetate-producing microorganisms, which may lead to the decrease in acetate / propionate ratios.

**Figure 7:**
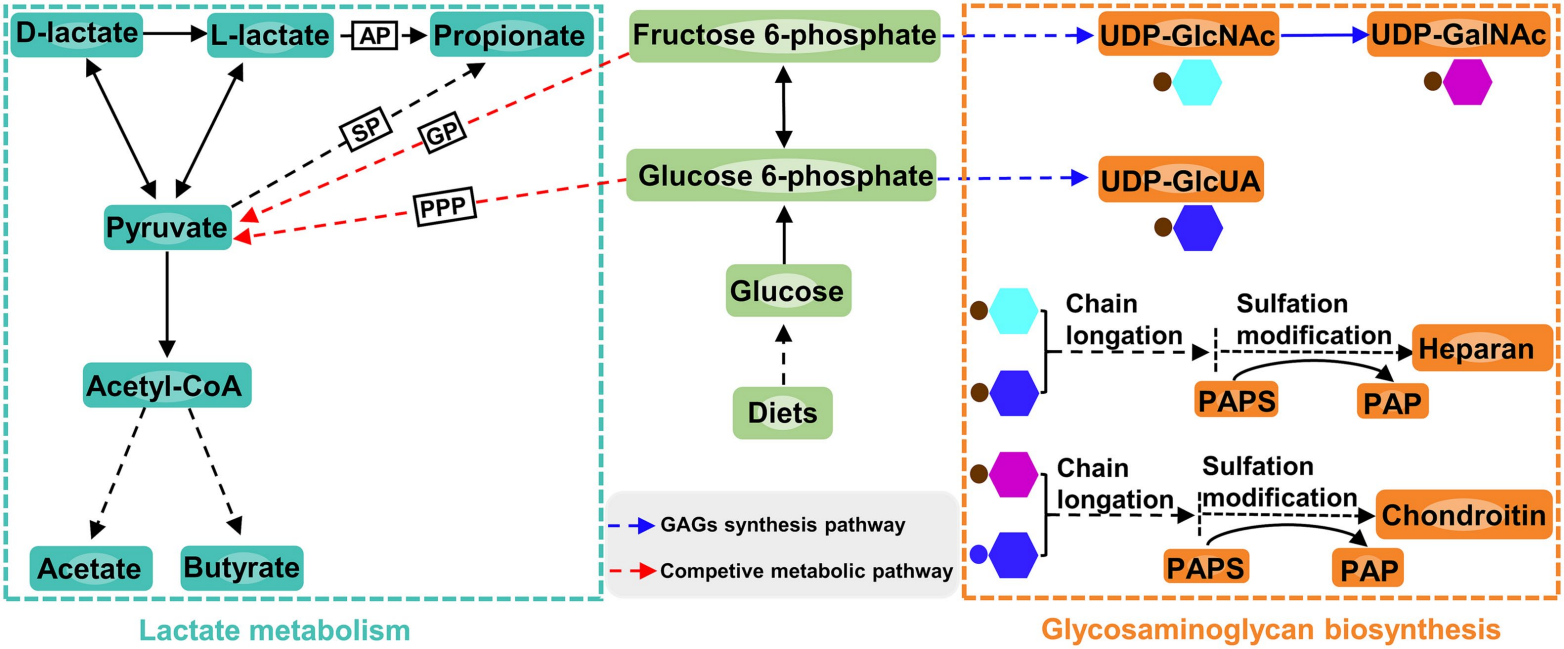
Lactate metabolism pathway, glucosaminoglycan biosynthesis pathway and the connection between the two pathways. AP, acrylate pathway; SP, succinate pathway; GP, glycolytic pathway; PPP, pentose phosphate pathway; GAG, glucosaminoglycan; UDP, uridine 5′-diphosphate; UDP-GlcNAc, UDP-N-acetylglucosamine; UDP-GalNAc, UDP-N-acetylgalactosamine; UDP-GlcUA, UDP-glucuronic acid; PAPS, 3′-phosphoadenosine-5′-phosphosulfate; PAP, 3′-phosphoadenosine-5′-phosphate.

Apart from affecting the composition of ruminal microbiota, increasing dietary *D*-lactate levels also altered the ruminal metabolism. In the current study, the glycosaminoglycan biosynthesis was closely related to *D*-lactate metabolism (Figure 7). The differential metabolite enriched in this metabolic pathway was 3′-phosphoadenosine-5′-phosphate (PAP), which was produced from sulfation modification of glycosaminoglycan chains by 3′-phosphoadenosine-5′-phosphosulfate (PAPS) (Figure 7). Correlation analysis showed that PAP was significantly negatively correlated with *Entodinium* and *Eubacterium_oxidoreducens_group*. *Entodinium* and *Eubacterium_oxidoreducens_group* could degrade fiber and starch to produce VFAs (Ivan et al., 2000; Lu et al., 2020). The increase in the products of glycolytic pathway such as VFAs could reduce the carbon flux toward glycosaminoglycan synthesis (Liu et al., 2023; Figure 7). Therefore, the glycosaminoglycan biosynthesis may be affected by the increase in the relative abundance of *Entodinium* and *Eubacterium_oxidoreducens_group*. Moreover, previous studies showed that chondroitin and heparin could inhibit the proliferation of pathogens such as *Escherichia coli* and reduce them infection of gastrointestinal epithelial cells (Gu et al., 2008; Chen et al., 2012; Unver et al., 2023). In this trail, the relative abundance of *Escherichia-Shigella* increased with the increase of dietary *D*-lactate levels. It could be inferred that the increase in the relative abundance of *Escherichia-Shigella* may be related to the alteration of the glycosaminoglycan (heparin and chondroitin) biosynthesis. It’s worth noting that the relative abundance of *Verrucomicrobiota* may be related to the alteration of glycosaminoglycan biosynthesis. *Verrucomicrobiota* contains glycoside hydrolases and sulfatases, which makes it regarded as a key degrader of glycosaminoglycan (Orellana et al., 2022). For example, Zhou et al. (2019) reported that feeding heparin could increase the relative abundance of *Verrucomicrobiota* in the mice. Accordingly, the alteration of glycosaminoglycan biosynthesis may in turn affect the relative abundance of *Verrucomicrobiota* and *Escherichia-Shigella*, reflecting the complex interaction between ruminal microorganisms and metabolites. The further research is required to clarify the causal relationship between the ruminal microbiota and metabolites.

## 5 Conclusion

In summary, increasing dietary *D*-lactate levels could increase VFAs concentrations and reduce acetate/propionate ratios, thus improving the energy supply of beef cattle. However, it also brought some negative effects, manifested as the increases in ruminal CH_4_ production and the relative abundance of *Escherichia-Shigella*. Multi-omics analysis demonstrated that increasing dietary *D*-lactate levels changed certain rumen microorganisms, resulting in the decrease in acetate/propionate ratios, the increase in CH_4_ and H_2_ production, and the alteration of glycosaminoglycan synthesis in the rumen. The current investigation not only facilitated our understanding of the influence mechanism of dietary *D*-lactate on rumen fermentation, but also provided scientific reference for the rational application of whole-plant corn silage in ruminant production. However, further investigation is needed on the metabolic process of dietary *D*-lactate in the rumen.

## Data availability statement

The datasets presented in this study can be found in online repositories. The names of the repository/repositories and accession number(s) can be found in the article/Supplementary material.

## Ethics statement

The animal studies were approved by Animal Care Committee, College of Animal Science and Technology, Hunan Agricultural University, Changsha, China. The studies were conducted in accordance with the local legislation and institutional requirements. Written informed consent was obtained from the owners for the participation of their animals in this study.

## Author contributions

QG: Investigation, Writing – original draft, Formal analysis, Methodology, Software, Visualization. JH: Investigation, Writing – review & editing. JW: Investigation, Writing – review & editing. YY: Investigation, Writing – review & editing. LL: Writing – review & editing, Formal analysis. ZW: Writing – review & editing, Data curation. WS: Conceptualization, Methodology, Validation, Writing – review & editing. FW: Conceptualization, Funding acquisition, Writing – review & editing, Formal analysis, Methodology.

## Footnotes

^1^ http://www.kegg.jp

^2^ http://www.metaboanalyst.ca/
