# Supplementary material for: Effects of dietary D-lactate levels on rumen fermentation, microflora and metabolomics of beef cattle
Source: Front Microbiol. 2024 Feb 6;15:1348729. doi: 10.3389/fmicb.2024.1348729 (PMC10877051; doi:10.3389/fmicb.2024.1348729)
Supplement: Supplementary file 1 [file Data_Sheet_1.pdf]

Tables

Table S1. Differential metabolites between the D-LA(0.3%) and D-LA(1.2%) groups.

Item	m/z	Rt/min	VIP	FC	Trend	*p*-value
Neohesperidin dihydrochalcone	611.18	37.52	2.37	0.03	↑	<0.01
Quercetin 3-(6-acetylglucoside)	505.10	58.37	2.30	0.10	↑	<0.01
Gravacridonol	324.13	37.85	2.41	0.13	↑	<0.01
3-Hydroxymethylglutaric acid	161.05	37.47	2.40	0.16	↑	<0.01
Aflatoxin GM1	345.06	38.10	2.24	0.17	↑	<0.01
LysoPC(14:0/0:0)	468.31	123.0	2.31	0.38	↑	<0.01
N-p-Coumaroyloctopamine	344.10	58.77	1.85	0.40	↑	<0.01
Citramalic acid	147.03	114.07	2.25	0.44	↑	<0.01
PE(16:0/20:0)	748.58	43.35	1.77	0.52	↑	<0.01
N4-Acetylcytidine	284.08	166.78	1.58	0.52	↑	0.02
Artonin K	383.10	108.34	2.09	0.53	↑	<0.01
Urolithin A-3-O-glucuronide	405.09	108.27	1.97	0.54	↑	<0.01
LysoPE(16:1/0:0)	452.28	123.87	2.11	0.5	↑	<0.01
LysoPE(15:0/0:0)	440.28	124.98	2.18	0.60	↑	<0.01
Deoxyadenosine	252.1	63.09	1.85	0.61	↑	<0.01
2-acetyl-1-alkyl-sn-glycero-3-phosphocholine	524.37	122.52	2.24	0.61	↑	<0.01
"1-Methylpyrrolo[1,2-a]pyrazine"	133.08	24.63	1.53	0.63	↑	0.03
Myristic acid	227.20	20.75	1.57	0.64	↑	0.03
Ribothymidine	257.08	38.82	2.18	1.52	↓	<0.01
3′-phosphoadenosine-5′-phosphate	428.04	260.18	1.64	1.56	↓	0.02
"1,5-Anhydrosorbitol"	163.06	26.196	1.95	1.61	↓	<0.01
"(S,E)-Zearalenone"	319.15	289.92	1.53	1.62	↓	0.03
"2,3-Dinor-TXB2"	341.20	52.42	2.02	1.67	↓	<0.01
Epinephrine	184.10	157.42	1.82	1.67	↓	<0.01
Dehydroxymethylflazine	279.08	58.55	1.42	1.71	↓	0.04
Gingerol	293.18	14.82	1.67	1.76	↓	0.02
4-Dodecylbenzenesulfonic acid	325.18	15.95	1.43	1.92	↓	0.04
2-Keto-6-acetamidocaproate	188.09	72.59	1.61	2.10	↓	0.02
4-Methylcatechol	169.05	54.93	1.98	2.46	↓	<0.01

m/z, mass-to-charge ratios; Rt, retention time; VIP, variable importance in projection; FC, fold change.

Table S2. Effects of dietary *D*-lactate levels on the rumen protozoan count of beef cattle.

Protozoan count/ $\times 10^5$	Dietary *D*-lactate levels, DM basis			SEM	*p*-value		
	0.3%	0.75%	1.2%		Treatment	Linear	Quadratic
Day 1	5.45	5.45	5.44	0.05	0.99	0.98	0.93
Day 2	2.44	2.22	2.37	0.05	0.23	0.57	0.11
Day 3	1.73	1.55	1.68	0.06	0.44	0.73	0.22
Day 4	1.12	1.03	1.17	0.05	0.51	0.69	0.28
Day 5	0.65	0.63	0.69	0.02	0.40	0.40	0.29
Day 6	0.67	0.61	0.60	0.19	0.23	0.11	0.60
Day 7	0.65	0.60	0.60	0.02	0.50	0.32	0.55
Day 8	0.55	0.57	0.53	0.01	0.35	0.63	0.18

SEM, standard error of the mean.

Figures

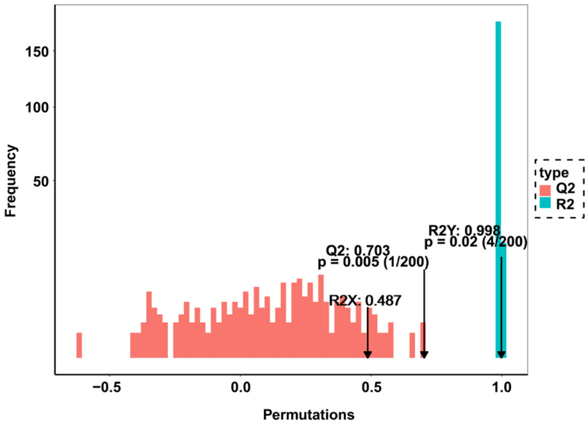

Figure S1. OPLS-DA model verification. R2Y represents the interpretation rate of Y matrix, Q2 represents the prediction ability of the model, and the closer the R2Y and Q2 values are to 1, the model is more reliable.
